# Multiple Immune-Inflammatory and Oxidative and Nitrosative Stress Pathways Explain the Frequent Presence of Depression in Multiple Sclerosis

**DOI:** 10.1007/s12035-017-0843-5

**Published:** 2018-01-02

**Authors:** Gerwyn Morris, Edna Maria Vissoci Reiche, Andrea Murru, André F. Carvalho, Michael Maes, Michael Berk, Basant K. Puri

**Affiliations:** 1 IMPACT Strategic Research Centre, School of Medicine, Deakin University, Barwon Health, Geelong, Australia; 2 Department of Pathology, Clinical Analysis, and Toxicology, Health Sciences Center, State University of Londrina, Londrina, Paraná, Brazil; 3 Bipolar Disorders Program, Hospital Clínic Barcelona, IDIBAPS, CIBERSAM, Barcelona, Spain; 4 Department of Clinical Medicine and Translational Psychiatry Research Group, Faculty of Medicine, Federal University of Ceará, Fortaleza, CE Brazil; 5 Department of Psychiatry, Faculty of Medicine, Chulalongkorn University, Bangkok, Thailand; 6 Department of Psychiatry, Medical University Plovdiv, Plovdiv, Bulgaria; 7 Department of Psychiatry, Faculty of Medicine, State University of Londrina, Londrina, Brazil; 8 Revitalis, Waalre, The Netherlands; 9 Orygen - The National Centre of Excellence in Youth Mental Health, The Department of Psychiatry and the Florey Institute for Neuroscience and Mental Health, The University of Melbourne, Parkville, Australia; 10 Department of Medicine, Imperial College London, Hammersmith Hospital, London, UK

**Keywords:** Depression, Multiple sclerosis, Immune function, Inflammation, Desipramine, Lofepramine, Oxidative and nitrosative stress

## Abstract

Patients with a diagnosis of multiple sclerosis (MS) or major depressive disorder (MDD) share a wide array of biological abnormalities which are increasingly considered to play a contributory role in the pathogenesis and pathophysiology of both illnesses. Shared abnormalities include peripheral inflammation, neuroinflammation, chronic oxidative and nitrosative stress, mitochondrial dysfunction, gut dysbiosis, increased intestinal barrier permeability with bacterial translocation into the systemic circulation, neuroendocrine abnormalities and microglial pathology. Patients with MS and MDD also display a wide range of neuroimaging abnormalities and patients with MS who display symptoms of depression present with different neuroimaging profiles compared with MS patients who are depression-free. The precise details of such pathology are markedly different however. The recruitment of activated encephalitogenic Th17 T cells and subsequent bidirectional interaction leading to classically activated microglia is now considered to lie at the core of MS-specific pathology. The presence of activated microglia is common to both illnesses although the pattern of such action throughout the brain appears to be different. Upregulation of miRNAs also appears to be involved in microglial neurotoxicity and indeed T cell pathology in MS but does not appear to play a major role in MDD. It is suggested that the antidepressant lofepramine, and in particular its active metabolite desipramine, may be beneficial not only for depressive symptomatology but also for the neurological symptoms of MS. One clinical trial has been carried out thus far with, in particular, promising MRI findings.

## Introduction

Multiple sclerosis (MS) is the most common inflammatory, demyelinating, neurodegenerative disease of the central nervous system (CNS) and is the leading source of non-traumatic neurological disability among young adults in Europe and North America [1, 2]. The wide array of core neurological symptoms associated with this illness includes sensory disturbances, limb weakness, optic neuritis, ataxia, profound bladder dysfunction, cognitive dysfunction, gastro-intestinal symptoms, fatigue and depression [2, 3]. Therefore, MS is viewed as a heterogeneous phenotype, and an expert consensus has proposed some discrete courses for this illness, including a clinically isolated syndrome, a relapsing-remitting disease, and progressive forms of the disease.

In 1887, Jean-Martin Charcot noted that a significant proportion of patients with MS display neuropsychiatric disturbances throughout the course of the illness. Since then, a significant body of evidence has accumulated and indicated that depressive symptoms represent the most prevalent of such neuropsychiatric disturbances seen in MS patients [4, 5]. The prevalence of depressive symptoms in MS patients has varied across studies. A systematic review and meta-analysis of population-based studies has estimated a prevalence of 23.7% (95% CI: 17.4–30.0%) [6], while prevalence rates seem to be substantially higher in specialised clinics (up to 50%) [4].

A growing body of evidence indicates that patients displaying symptoms of depression have a worse prognosis than those who do not. It is noteworthy that prospective data indicate that mood symptoms fluctuate over the course of MS and that the advent of depressive symptoms is often associated with disease relapse.

The presence of depressive symptoms is predictive of an impaired quality of life and such symptoms can either co-exist with severe fatigue and/or significant cognitive impairment or occur independently. Cognitive impairment may affect up to 60% of patients with MS [7–9] and the severity of depressive symptoms correlates with increased impairment in executive function and processing speed [10, 11]. Depression also is a significant contributor to the higher suicide rates observed among individuals with MS, with the standardised mortality ratio for suicide in such patients being around twice as high as that in the general population [12].

Major depressive disorder (MDD) is increasingly regarded as a unitary and very serious neuroprogressive illness with a remitting-relapsing pattern leading to major disability and reduction in life quality together with increased cardiovascular and other significant medical morbidity [13]. This illness is currently estimated to be the fourth leading cause of serious disability in the world and is projected to become the second leading cause of disabling morbidity worldwide by 2020 [14]. The question arises as to whether the high rates of depressive symptoms seen in MS patients represent the same condition as MDD and is the assumption that some patients with MS also have MDD as a separate illness correct or are such symptoms a manifestation of the core disease processes driving the pathogenesis and pathophysiology of the latter illness [15]. In this context, it is intriguing that there is accumulating evidence suggesting that there may be some common elements underpinning the pathogenesis and pathophysiology of MDD and MS. For example, it is noteworthy that neuroinflammation and neurodegeneration are both considered to be crucial elements in the pathogenesis and pathophysiology of both MS and MDD [16–18]. The weight of evidence also indicates that this is also true of peripheral inflammation as evidenced by elevated levels of proinflammatory cytokines (PICs), acute-phase proteins, such as C-reactive protein (CRP), and transcriptional factors, such as nuclear factor kappa B (NF-κB) [19, 20]. Indeed, increased rates of peripheral inflammation, notably interleukin (IL)-6 and CRP, appear to differentiate MS patients suffering from comorbid depression from those who do not [21]. Interestingly, there is also some evidence to suggest patients reporting symptoms of depression also present with more advanced disease and increased clinical disability, as measured by the Expanded Disability Status Scale (EDSS), compared with their depression-free counterparts, although whether these findings indicate that depressive symptoms emanate from core disease processes or whether the advent of MDD is an independent driver of pathology remains an open question [21].

MS and MDD patients share many other abnormalities, such as chronic oxidative and nitrosative stress (O&NS) characterised by oxidatively and nitrosatively damaged proteins, lipids and DNA, and consequent loss of immunogenic tolerance [22]. Given the strong association between chronic oxidative stress and the development of mitochondrial dysfunction, the presence of widespread damage to this organelle in both illnesses is unsurprising [19, 20]. Impaired natural killer (NK) cell function and/or reduced numbers of these lymphocytes are also characteristic features of MDD and MS [23, 24]. Disturbed T cell homeostasis and recruitment of myelin autoreactive encephalogenic T cells into the CNS is still considered to be the ultimate driver of progressive pathology in MS, but there is also a growing awareness that impaired T cell homeostasis and recruitment into the CNS may well play an important role in the pathophysiology of MDD [17]. Further evidence of impaired T cell homeostasis in both illnesses is provided by several research groups who have reported abnormalities in function and/or number of regulatory T cells (Tregs) [25, 26]. Functional polymorphisms affecting cytokine production and/or function and T cell homeostasis and activation increase the risk of developing both illnesses [27–29].

Epigenetic dysregulation is also seen in MS and MDD with widespread changes in DNA methylation, histone acetylation and microRNA (miRNA) transcription leading to the abnormal expression of a multitude of genes, although the precise pattern of altered gene expression is markedly different [30, 31]. However, despite copious research, the mechanisms underpinning the pathogenesis of both illnesses remain to be fully delineated and the contributions of many of the abnormalities to disease pathology are still the subject of some controversy. The situation is made more complex by evidence indicating that MDD and MS are very likely pathologically heterogeneous conditions [32, 33]. While there is a growing consensus that inflammation in the periphery and the brain together with oxidative stress and mitochondrial dysfunction play a role in the aetiopathogenesis of both illnesses, there are major differences in abnormalities recorded in many other domains such as T cell dysfunction [16, 18, 19].

The precise biological mechanisms underpinning the existence of depression in MS and their importance in contributing to the pathophysiology of MS are still the subject of some debate. In addition, as previously indicated, the issue remains outstanding as to whether depressive symptomology accompanying MS can be regarded either as evidence of the existence of MDD as a separate condition acting as a driver of pathology or be a product of core disease processes. In this paper, we aim to compare findings in MS and MDD produced by various research teams investigating immune abnormalities, glial cell activity, blood brain barrier permeability [34], autoimmune manifestations, impaired gastrointestinal epithelial barrier permeability, activity of inflammatory pathways, neuroimaging and bioenergetics in order to comment on the likely answer or answers to this question or indeed whether such a dichotomous viewpoint is appropriate. Finally, we suggest translational implications for this expanding body of knowledge.

## Methods

Pursuant to our interest in comparing and contrasting a wide range of biological abnormalities in MS and MDD, a search of MEDLINE and Web of Science was undertaken with keyword inputs of MS, depression, T cell, B cell, oxidative stress, nitrosative tress, nitric oxide (NO), mitochondrial dysfunction, inflammation, immune activation, microglia, astrocytes, neuroinflammation, neoepitopes, autoimmunity, antioxidants, antioxidant therapy, gut dysbiosis, increased intestinal permeability, leaky gut, bacterial translocation, epigenetic, histone acetylation, DNA methylation and microRNA.

## Evidence of O&NS in MS and MDD

### O&NS in MS

There are multiple lines of evidence demonstrating that chronic O&NS plays a major causative role in the pathogenesis of MS [18, 19, 35]. Such evidence includes data demonstrating dramatic increases in O&NS levels during relapse, which falls to virtually undetectable levels when patients enter into remission [36]. Levels of O&NS also correlate with the extent of clinical disability as ascertained by the EDSS score and the extent of gadolinium-enhanced lesions observed with structural magnetic resonance imaging (MRI) scanning [37, 38]. Furthermore, Tasset and fellow workers have reported significant peripheral levels of oxidative stress in patients with relapsing-remitting MS (RRMS) [39, 40]. Several research teams have reported the presence of O&NS damage to proteins and lipids, such as elevated levels of protein carbonyls in the brain, cerebrospinal fluid (CSF) and peripheral blood of MS patients in vivo and in post mortem studies [19, 41, 42]. Several other surrogate markers of O&NS and lipid peroxidation are frequently observed in the CSF and plasma of MS patients [42, 43], with the presence of malondialdehyde (MDA) [44], hydroxynonenal [41] and isoprostanes [45] being the most common findings. Interestingly, and perhaps predictably, levels of NO metabolites in the CSF correlate positively with the number of relapses suffered by a patient over a given period [46]. Unsurprisingly, significantly increased levels of NO, peroxynitrite and superoxide are also present in spinal fluid extracts [44] and high levels of nitrotyrosine, indicating the presence of peroxynitrite and inducible NO synthase (iNOS) are often reported in active demyelinated white matter lesions [44, 47].

### O&NS in MDD

O&NS, resulting from increased reactive oxygen species (ROS), reactive nitrogen species (RNS) and compromised cellular antioxidant systems, is also considered to be a major factor driving the pathophysiology of MDD [48–50]. Evidence of compromised antioxidant system activity includes elevated levels of superoxide dismutase and catalase activity [51] and deficiencies in levels of antioxidant molecules, such as vitamins C and E [52–54]. Several authors have also reported the presence of widespread lipid peroxidation in the prefrontal cortex and other crucial areas of the brain linked to the development of MDD [55, 56]. Interestingly, lipid peroxidation levels correlate with symptom severity in females but not in males for reasons which remain to be delineated [55, 56]. The presence of lipid peroxidation is reinforced by data demonstrating the existence of increased MDA concentrations in serum and extensive oxidative damage to a wide range of lipids in peripheral tissues [51, 57]. Several research teams have also reported increased levels of isoprostane and 8-oxo-2′-deoxyguanosine in urine and plasma samples, bearing further testimony to the presence of high ROS and RNS levels produced outside the brain in patients suffering from depression [52–54]. In this context, it is noteworthy that the levels of peripheral oxidative stress markers correlate with severity and chronicity in both male and female patients [50, 56–58].

Given this relationship, the presence of a large number of papers reporting the results of trials into the efficacy of antioxidant compounds as potential antidepressant treatments is unsurprising [59, 60]. Furthermore, the extent of oxidative damage to proteins, lipids and DNA is of sufficient magnitude as to form neoepitopes owing to loss of immunological tolerance leading to autoantibody responses and the development of autoimmunity [49, 61, 62]. The presence of oxidative damage to mitochondrial DNA (mtDNA) observed in patients suffering from MDD is further evidence of the severity of peripheral nitroxidative stress associated with this illness [63, 64].

## Evidence of Lowered Antioxidant Levels

### Antioxidants in MS

There is ample evidence of lowered serum total antioxidant capacity in MS patients with reduced levels of plasma albumin ascorbate, alpha tocopherol, retinol urate and bilirubin compared with healthy controls being repeatedly reported [65–67]. Altered glutathione homeostasis, especially reduced glutathione reductase and glutathione peroxidase, is also a well-documented phenomenon although some research teams have not observed such abnormalities in their study cohorts [68, 69]. The activity of the thioredoxin system is also compromised in at least some patients diagnosed with this illness with increased thioredoxin activity but impaired thioredoxin reductase activity being the most commonly reported findings [70]. Superoxide dismutase and catalase activities are increased in grey matter, microglia, lesions and the CSF, but impaired in peripheral blood mononuclear cells (PBMCs) and erythrocytes in the periphery [71–74]. Finally, there are several studies reporting increased activity of the transcription factor nuclear factor (erythroid-derived 2)-like 2 (NFE2L2 or Nrf2) and of antioxidant response elements (ARE) in the brain and periphery but that this increased activity is unable to prevent the oxidative damage to molecules in the environment of chronically increased ROS and RNS seen in MS patients [70, 75, 76].

Several antioxidant regimes have demonstrated clinical and neurological benefit in non-human animal models of MS but results in humans, in whom very high doses of antioxidants are needed to achieve functional effects in the CNS, have been relatively disappointing until relatively recently. However, activators of Nrf2-ARE signalling pathway such as dimethyl fumarate (DMF) are now showing considerable promise [77, 78]. Activation of this pathway has several beneficial consequences, such as activation of the haem oxygen pathway and increased synthesis of reduced glutathione, inhibition of NF-κB and PIC production, and a concomitant provocation of a Th1 to Th2 lymphocyte shift [79, 80]. The authors of the CONFIRM and DEFINE studies were the first to report a significant decrease in the annual relapse rate, reductions in the number of gadolinium-enhanced MRI lesions and new or hyper-intense legions in patients treated with DMF compared with interferon (IFN)-β [81, 82]. These findings have been confirmed by several other research teams [83–85]. MitoQ has been successful in delaying progression and reducing neurological disability in experimental autoimmune encephalomyelitis (EAE) [86] and standard formulations of coenzyme Q_10_ (CoQ10) at 500 mg/day have demonstrated a capacity to reduce inflammatory markers in people with MS [87]. However, there are no studies demonstrating any benefit on neuroimaging indices or clinical disability although CoQ10 at 500 mg/day appears to produce a significant reduction in the symptoms of fatigue and depression in MS patients [88]. Polyunsaturated fatty acids (PUFAs) are another class of nutrients which have shown clinical and neurological improvement in some MS patients albeit at very high doses for prolonged periods of time [89]. However, while PUFAs clearly have antioxidant effects, their mode of action is multifactorial and these entities affect very similar pathways to those influenced by DMF [90].

### Antioxidant Levels in MDD

MDD patients characteristically display significantly lower serum concentrations of a wide array of crucial antioxidants, such as zinc, ascorbic acid and CoQ10 [91]. Several research teams have also reported lower plasma total antioxidant status compared with unaffected controls [92–94]. There is also evidence of impaired activity of the glutathione system with reduced levels of reduced, oxidised and total glutathione reported in the post mortem brains of MDD patients [94]. The concentration and activity of key enzymes within the glutathione system, namely glutathione peroxidase and glutathione-S-transferase, also appear to be reduced in the brain and periphery of patients suffering from this illness [95, 96]. While there is limited published work investigating the status of the thioredoxin system in MDD patients [97], there is ample evidence of abnormal MnSOD and catalase activity [98–100]. Catalase activity appears to be consistently increased but the data for MnSOD are somewhat inconsistent with reports of both increased serum activity [98, 99] and decreased serum activity [100]. There is no direct evidence of abnormal Nrf2 or DJ-1 (PARK7) activity in MDD patients although there is some evidence from animal studies suggesting that the expression of Nrf2 may be reduced [101].

While several classes of antioxidants have proven to be efficacious in treating depression in animal models of the illness, the overall results in human studies have been rather disappointing especially as far as unipolar depression is concerned (review [92, 102]). There are some data demonstrating that 1200 mg CoQ10 per day over 8 weeks can significantly reduce depressive symptoms in patients with bipolar disorder [103, 104] and a recent study by Gaultan and others demonstrated a significant treatment effect in unipolar depression using vitamins C (in the form of ascorbic acid), E (as alpha-tocopherol) and A (as beta-carotene) over a 6-week period [105]. A meta-analysis of 241 studies concluded that supplements containing eicosapentaenoic acid (EPA) and docosahexaenoic acid appeared to convey significant benefit in treating MDD if the EPA content was greater than 50% of the total concentration [106]. A reader interested in a more detailed examination of these studies is invited to consult a review by [89]. Therapeutic administration of *N*-acetylcysteine has demonstrated therapeutic benefit in a number of illnesses in which chronic inflammation and oxidative stress play a major pathogenic role (reviewed [89]). Disappointingly, it only demonstrated slight non-significant effects on symptom reduction in MDD [59], although the doses used in this trial were significantly lower than the doses proven to convey therapeutic benefit in other inflammatory illnesses [18]. Meta analyses of *N*-acetylcysteine however seem to show a positive signal [107]. The use of polyphenols such as apigenin, amentoflavone, chlorogenic acid, ferulic acid, resveratrol and curcumin have recently attracted a great deal of attraction following their success in non-human animal studies and their broad range of positive effects on inflammation, oxidative stress, mitochondrial dysfunction, intestinal permeability and serotoninergic and dopaminergic neurotransmission [108, 109]. Lopresti and colleagues reported a significant improvement in symptoms in patients with MDD following 1000 mg/day of curcumin, particularly those patients suffering from depression with atypical features [108]. However, replication of these results in larger samples is due before definitive conclusions can be made regarding the therapeutic benefit of this supplement. A summary of similarities and differences between MS and MDD in the redox domain is provided by Table 1.

**Table 1:** Similarities in O&NS pathways and antioxidant levels between MS and MDD

O&NS	MS	MDD
Lipid peroxidation	Y	Y
Increased malondialdehyde	Y	Y
Elevated peroxynitrite	Y	Y
Nitrated amino acids	Y	Y
Elevated NO	Y	Y
Elevated iNOS	Y	Y
Raised isoprostane levels	Y	Y
Low vitamin E	N	Y
Reduced levels of glutathione	Y	Y
Low zinc levels	N	Y
Low CoQ10 concentrations	N	Y
O&NS implicated in pathology	Y	Y

## Evidence of Peripheral Inflammation in MS and MDD

### Peripheral Inflammation in MS

There is now copious evidence of the distribution patterns and levels of a wide range of PICs and anti-inflammatory cytokines in the CSF, post mortem brains and serum of patients diagnosed with RRMS and indeed all other presentations of the disease [110–112]. Examples of elevated PICs include IFN-γ, tumour necrosis factor (TNF)-α, IL-12 IL-1β, IL-6, IL-15, IL-4, IL-17, IL-10, transforming growth factor (TGF)-β1, IL-27 and IL-23 [112, 113]. It is noteworthy that the overall pattern of cytokine abnormalities seen in patients in the active phase of the illness differs from that characteristically observed in patients who have entered remission [106, 109]. In particular, elevated levels of TGF-β1 and IL-10 are characteristic of the latter individuals while elevated levels of PICs, most notably TNF-α, IL-1β and IFN-γ, are characteristic of patients following relapse [110, 113].

Importantly, increasing levels of TNF-α in the peripheral circulation are predictive of relapse and levels of this PIC correlate with clinical disability estimated by EDSS scores [110, 114, 115]. Levels of TNF-α correlate with the severity of fatigue experienced by many MS patients, which has a major detrimental impact on their quality of life [116]. It is also noteworthy that functional single nucleotide polymorphisms (SNPs) in several genes encoding TNF superfamily members would appear to be the largest non-human leukocyte antigen (HLA) risk factors for developing the illness with the *TNF-β Nco*l polymorphism rs909253, in the first intron of *TNF-β*, conferring the greatest risk [113, 114]. There is an accumulating body of evidence demonstrating that this SNP combined with elevated O&NS is associated with a marked increase in disease progression and levels of clinical disability and unsurprisingly the *TNF-β Nco*l polymorphism rs909253 continues to be the focus of intense research [117, 118].

Levels of IL-6, CRP and other markers of systemic inflammation appear to be higher in MS patients reporting symptoms of depression compared with patients who do not report such symptoms [21]. It is also noteworthy that elevated levels of IL-6 are also associated with increased symptoms and increased severity of disease experienced by MS patients exposed to protracted periods of extreme psychosocial stress [119]. IL-1β is another PIC implicated in the pathogenesis and pathophysiology of MS [116, 117]. In particular, the presence of this cytokine in the CSF of patients currently in remission foretells a poor prognosis and the development of a more aggressive disease profile as determined by EDSS [120]. In addition, detectable levels of IL-1β observed in the CSF of newly diagnosed treatment-naïve patients are an invariant marker of significant brain damage and correlate positively with cortical lesion volume [121].

Elevated CNS and serum levels of IL-17 are also commonly detected in MS patients [122, 123]. The multiple pathological effects of this cytokine include direct cellular and tissue damage as well as promotion of the maturation of dendritic cells and increased production of TNF-α [122]. The contribution made by IL-17 to the pathogenesis of RRMS is highlighted by data demonstrating that levels of IL-17 correlate with the frequency of relapses endured by a patient and are strongly predictive of the failure of IFN-β therapy [122, 123]. The ultimate origin of cytokine abnormalities in MS appear to be activated T and B cells in the periphery while activated microglia B lymphocytes and monocytes are the major source of cytokines in the CSF [124].

Activation of the nucleotide-binding oligomerisation domain (NOD)-like receptor (NLR) family, pyrin domain containing 3 (NLRP3) inflammasome, also appears to be a source of PIC production, and the weight of evidence suggests that activated dendritic cells make an additional contribution [125, 126]. However, despite copious evidence implicating the pathogenic role of PICs and IL-17 in the development of RRMS, there are also data indicating that anti-inflammatory cytokines are drivers of pathology in some patients [127].

### Peripheral Inflammation in MDD

There is a developing consensus which places chronic systemic inflammation, as evidenced by elevated PICs and/or a dysregulated pattern of cytokine production, as the ultimate driver of major depression [17, 128–130]. It is noteworthy that increased levels of peripheral PICs in combination with increased free ROS and RNS production go some way towards explaining the increased risk of developing cardiovascular, metabolic and other serious medical diseases in patients afforded a diagnosis of MDD compared with age- and sex-matched controls [131, 132]. Several meta-analyses have revealed that increased levels of Il-6 and TNF-α would seem to be the most consistent findings in MDD patients, but elevated levels of IFN-γ and IL-1β are also frequently reported [13, 133, 134]. The importance of PICs in the pathogenesis of MDD has been emphasised by recent evidence published by Young and fellow workers, who reported that the magnitude of peripheral PIC elevation correlated with the progression and severity of the disease [135]. This conclusion supports the work of earlier research teams who reported a correlation between levels of PICs in the CSF of MDD patients and the severity of symptoms they endured [26]. Moreover, Maes and fellow workers have established an association between the presence of functional SNPs in *TNF-α*, *IL-1β* and *CRP* and an increased risk of developing depression and, indeed, responsiveness or otherwise to the administration of antidepressant therapy [94].

The origin of PICs in MDD is thought to be M1 (classically activated) polarised macrophages and, possibly, neutrophils but there is also accumulating evidence of activated, if anergic, T cells in at least some MDD patients [94]. The activation of the NLRP3 inflammasome by danger-associated molecular patterns (DAMPs) (also known as damage-associated molecular patterns), such as heat-shock protein-72 (HSP72), mtDNA and uric acid, leading to the increased production of activated IL-1β and IL-18, may also be involved [27]. In the context of examining potential sources of inflammation in MDD patients, it is noteworthy that DAMP-mediated activation of the NLRP3 inflammasome is potentiated by the presence of commensal lipopolysaccharide (LPS) translocated into the peripheral circulation as a result of increased intestinal permeability [136, 137], Translocated LPS is also a major cause of Toll-like receptor (TLR) activation on antigen-presenting cells (APCs) and hence a potential source of T cell activation in MDD [17, 137]. Readers interested in a detailed treatment of this area are referred to a comprehensive review by Lucas and colleagues [138].

This would seem to be an appropriate juncture to add words of caution, as while elevated PICs would appear to be major drivers of symptoms in MDD patients, this is not invariably so. It would appear that, in at least some patients, the symptoms are associated with increased levels of anti-inflammatory cytokines, notably IL-4, IL-13 and IL-15, and the recovery is associated with increased levels of PICs, such as TNF-α [139]. This may reflect the fact that MDD is considered by many to be a biologically heterogeneous illness, as proposed by the latter authors, but there may also be other explanations. One such would be activation of the haem-containing enzyme indoleamine 2,3-dioxygenase (IDO) by PICs or LPS and a pattern of immunosuppression following the subsequent increase in levels of the L-tryptophan metabolite kynurenine [91]. It is also conceivable that direct Treg activation by LPS could provoke a Th2-biased cytokine pattern [140]. Peripheral cytokines can access the CNS to provoke activation of microglia and astrocytes via several mechanisms, such as via the circumventricular organs, which lack a fully functional blood-brain barrier (BBB) [18]. This influx of peripheral PICs leads to activation of perivascular macrophages and endothelial cells, which in turn secrete cytokines and other inflammatory mediators, such as a range of chemokines, NO and prostaglandin E2 (PGE2) [17, 141]. The other major mechanisms involved in relaying cytokine signalling to the CNS involve the stimulation of vagal nerve afferents in the spleen conveying cytokine signals to brain regions such as the hypothalamus and are often described as the neural route [142]. We now move to a consideration of the similarities and differences of the patterns of microglial pathology in MS and MDD. A summary of similarities and differences between MS and MDD regarding abnormalities in immune and inflammatory pathways is provided in Table 2.

**Table 2:** Similarities and differences in immune-inflammatory biomarkers between MS and MDD

Immuno-inflammatory pathways	MS	MDD
Raised levels of PICs	Y	Y
Increased NF-κB	Y	Y
Increased COX-2	Y	Y
Raised IL-2	Y	Y
Raised IL-10	N	Y
Raised TGF-β	Y	N
Co-existence of a Th1 and Th2 response	Y	Y
Presence of IL-17-secreting Th17 T cells	Y	Y
Temporal variation in cytokine profile	Y	Y
Astrocyte activation and or dysfunction	Y	Y
Treg dysfunction	Y	Y
FOXP3 dysfunction	Y	?
Clonal exhaustion of T cells	N	Y
Activated microglia and macrophages	Y	Y
Intrathecal IgG production	Y	N
Low NK cell activity	Y	Y
Chronic activation of immuno-inflammatory pathways	Y	Y

## Evidence of Microglial Pathology in MS and MDD

### Microglial Pathology in MS

Several authors have reported the presence of activated microglia expressing the cell surface receptor HLA-antigen D-related (HLA-DR), which (with its ligand) is a T cell receptor ligand, in the normal-appearing white matter (NAWM) of RRMS patients in the earliest stages of the disease even in the absence of BBB disruption [143, 144]. It is also noteworthy that the extent of microglial activation in the later stages of RRMS is associated with a worse prognosis and also correlates positively with the level of disability as ascertained by the EDSS score [145, 146]. Microglial actions in MS lesions can be dichotomised into the activities of M1 and M2 (alternatively activated) microglia (sometimes respectively designated as neurotoxic and neuroprotective). Neuroprotective microglia carry out their benign role by secreting a range of neurotrophic factors and also play a major role in aiding clearance of myelin and other debris and thus can be considered to have an anti-inflammatory and calming effect within the CNS [147]. Neurotoxic microglia, on the other hand, can provoke and accelerate the development of neuroinflammation via the secretion of PICs, ROS, RNS, PGE2, cyclooxygenase-2 (COX-2), quinolinic acid and glutamate [20, 148, 149]. Importantly, from the perspective of the pathogenesis and the pathophysiology of the disease, the polarisation of these glial cells into neurotoxic or neuroprotective phenotypes is heavily influenced and perhaps even determined by the environmental balance of microRNAs (miRNAs) in the environment, with increases in miR-689, miR-124 and miR-155 being associated with the development of an M1 phenotype while miR-124, miR-711 and miR-145 appear to mediate polarisation into an M2 phenotype [150]. In this context, it is important to note that the production of miR-155 is upregulated in microglia of RRMS patients and is a recognised driver of PIC synthesis and secretion [151]. The mechanisms underpinning these observations have yet to be fully delineated but it is noteworthy that there are data indicating that the transcription of mi-155 and mi-145 is responsive to levels of NF-κB, PICs and Nrf2 [152, 153]. Microglia also have the capacity to act as APCs, thereby activating naïve autoreactive T cells recruited from the peripheral circulation [146]. The recruitment of T cells from the periphery appears to play a crucial role in the pathogenesis and pathophysiology of RRMS and determines the pathological or benign consequences of microglial activation [154]. In particular, the influx of inflammatory encephalitogenic CD4^+^ T cells, including Th17, Th1 and γδT-cells, robustly promote the development of neuroinflammatory and neurodegenerative processes while recruitment of Tregs and Th2 ameliorate neuroinflammation stemming from microglial activation thereby playing a major role in the development of an anti-inflammatory and neuro-supportive environment [155, 156].

### Microglial Pathology in MDD

Several research teams have also reported the existence of activated microglia in patients afforded a diagnosis of MDD [157, 158]. Sustained activation of these glial cells would appear to be the ultimate source of the neuroinflammation seen in most, but by no means all, MDD patients [157, 159]. The weight of evidence implicating the presence of activated microglia as a causative factor in the pathogenesis and pathophysiology of MDD is accumulating so rapidly that one team of researchers has even described idiopathic MDD as “a microglial disease” [160]. The putative role of activated microglia in the initiation and progression of the illness is further reinforced by the existence of data demonstrating that the severity of symptoms experienced by MDD patients correlates with levels of quinolinic acid, which is a neurotoxin secreted by these glial cells in an activated state [161], and that the activation of microglia underpins the development of *N*-methyl-D-aspartate (NMDA) receptor excitotoxicity [158]. It is also of interest that many, if not all, antidepressants modulate the function and morphology of microglia and this property may well underpin their therapeutic effects seen in some patients [160]. However, there appear to be significant differences in the pattern of microglial activation in the hippocampus in MDD patients compared with the pattern seen in RRMS patients. Microglial pathology in MS is described in the previous subsection, while in the case of MDD the initial activation and proliferation of these glial cells is replaced by cell death in the form of apoptosis and/or a myriad of morphological changes which include reduced expression of a wide array of essential functional proteins indicative of profound dystrophy [162].

Interestingly, the development of dystrophy appears to be preventable in animal models of MDD by the early administration of the tetracycline-class antibiotic minocycline [162], which also demonstrates early efficacy signals in depression [163, 164]. Abnormalities in miRNA expression patterns have also been reported in the brains of MDD patients, but once again, the overall pattern seems to be significantly different from that detected in RRMS patients. Specifically, in the case of abnormalities in their levels, miRNA species appear focused on those playing a major role in modulating and enabling the many “housekeeping” roles of microglia in the CNS such as maintaining neuroplasticity, regulating long-term potentials involved in the development and consolidation of learning and memory, and the global performance of innate and adaptive immune signalling pathways within the brain [157]. There is some evidence of increased recruitment of Tregs into the CNS in MDD [26, 165]. This phenomenon may be of importance given data demonstrating that Tregs reduce levels of microglial activation and thus act to restrain the development of neuroinflammation [151, 152]. This would appear to be an important point as the recruitment of Tregs into the CNS in “idiopathic” MDD versus the pattern of Th17 and Th1 T cell recruitment into the CNS in RRMS may, at least in part, explain the different patterns of microglial pathology seen in each condition.

## Evidence of Mitochondrial Dysfunction in MS and MDD

### Mitochondrial Dysfunction in MS

While the weight of evidence indicates that inflammation and O&NS are the prime drivers of pathology in early MS, the ultimate trajectory of disease progression is heavily influenced by mitochondrial dysfunction and impaired production of adenosine triphosphate (ATP) in the brain and periphery [166, 167]. The range of mitochondrial abnormalities observed in MS includes altered cellular distribution and structure together with a wide range of biochemical and molecular abnormalities [20, 168–170]. Impaired activity of Complex I (reduced nicotinamide adenine dinucleotide (NADH)-Q oxidoreductase) of the electron transport chain (ETC) and extensive oxidative damage to mtDNA are characteristically observed in active MS lesions [171], while the activities of Complex I, Complex III (Q-cytochrome *c* oxidoreductase), and Complex IV (cytochrome *c* oxidase) are also impaired in NAWM of the motor cortex and elsewhere [172–174]. Complex IV activity is also decreased in normal-appearing grey matter (NAGM) as well as in white matter hyperintensities [173, 174].

Several authors using neurospectroscopy have reported the presence of lactate in the CSF of MS patients and a range of abnormalities consistent with globally impaired bioenergetics [175, 176]. A recent longitudinal study conducted by Lazzarino and colleagues provided support for the causative role of mitochondrial dysfunction in the pathophysiology of MS when they reported that progressive depletion of central ATP levels correlated with increased physical disability as measured by EDSS changes over a 3-year period [177]. Even more recently, a different group reported that not only did RRMS and secondary progressive MS (SPMS) patients have higher phosphocreatine (PCr) and lower phosphodiesters than age-matched healthy controls, but a higher β-ATP level was found in RRMS patients than in SPMS patients, with its level correlating negatively with the EDSS score in all the MS patients; these findings suggest a higher level of energy production in the former MS group, which might be related to an increased energy need than in the SPMS group [178].

*N*-acetylaspartate (NAA) levels can be measured in the brain using proton neurospectroscopy and is of particular interest because of its neuronal localisation [179]. Using a combination of diffusion tensor imaging and proton neurospectroscopy, in 2012 Wood and colleagues reported lower NAA parallel diffusivity (λ _‖_) in MS patients compared with age- and sex-matched controls [180]. They also found that NAA λ _‖_ was inversely correlated with water λ _‖_ and with clinical severity [174]. These results were consistent with axonal degeneration in MS. Five years later, the same group published the results of a six-month diffusion-weighted proton neurospectroscopy study in MS, reporting a decrease in NAA diffusivity in patients with evidence of inflammatory activity but not in those with neuroradiological and clinical stability [181]. Thus, this technique can be used to assess the downstream effects of acute inflammatory demyelination [175].

### Mitochondrial Dysfunction in MDD

There is also copious evidence demonstrating that mitochondrial dysfunction plays a causative role in the pathophysiology of MDD [58, 182]. This is perhaps unsurprising given the heavy involvement of O&NS in driving the genesis, persistence and severity of the illness and the fact that the bidirectional association between chronically elevated ROS and RNS levels and mitochondrial dysfunction has been conclusively established [13, 20, 149, 183]. The core symptoms of MDD, such as loss of motivation, lethargy, fatigue, neurocognitive impairment and sleep disturbances appear to be driven at least in part by dysfunction of enzyme clusters involved in the ETC [184, 185]. In addition, several authors have reported evidence indicating globally impaired bioenergetic metabolism in the brain of MDD patients, which is particularly deficient in the prefrontal cortices and the basal ganglia [184, 186]. Other research teams have detected global abnormalities in glucose metabolism, and a reliance on glycolysis as the major source of ATP generation [185, 187, 188]. Crucially, there are also a number of studies demonstrating the existence of profound mitochondrial dysfunction in peripheral immune cells and in a range of other tissues extracted from MDD patients [189, 190]. For example, Gardner and fellow workers reported mitochondrial dysfunction notably in Complexes III and IV of the ETC leading to impaired ATP generation in the striated muscle of MDD patients complaining of physical symptoms [191]. The presence of mitochondrial dysfunction in PBMCs of MDD patients has also recently been confirmed [192]. It is also noteworthy that impaired PBMC mitochondrial respiration correlates with the severity of many core MDD symptoms, notably fatigue, loss of energy and concentration difficulties, further supporting a causative role for a shortfall in ATP production as an important driver of such symptoms [192]. A summary of the similarities and differences between MS and MDD in the area of compromised bioenergetics is provided in Table 3.

**Table 3:** Comparison of mitochondrial dysfunction between MS and MDD

Mitochondrial dysfunction	MS	MDD
Depleted ATP production (muscle and brain)	Y	Y
Decreased PCr re-synthesis following exercise	Y	N
Impaired oxidative phosphorylation	Y	Y
Acceleration of glycolysis	Y	Y
Damage to mitochondrial respiratory chain	Y	Y
Oxidative mitochondrial damage	Y	Y
Mitochondrial energy failure	Y	Y

## Bacterial Translocation and Leaky Gut

### Leaky Gut in MS

Geffard’s group has established increased IgA and IgM responses to bacterial antigens and LPS in MS patients compared with controls [193, 194]. This indicates increased IgA- and IgM-mediated immune responses to Gram-negative commensal bacteria in the peripheral blood of patients with MS. There is some evidence that this phenomenon is probably a consequence of increased gut permeability in those patients.

Miyake and colleagues compared the intestinal microbiome of RRMS patients with that present in healthy participants, utilising next-generation pyrosequencing and culture-independent amplification of the bacterial 16S ribosomal RNA (rRNA) gene [195]. Their subsequent analysis revealed the presence 21 species of *Clostridia* and *Bacteroidetes* in MS patients which were not present in healthy controls. Moreover, there were significant differences within *Clostridia* clusters XIVa and IV and *Bacteroidetes* between RRMS patients and controls with a relative paucity in the *Clostridia* species normally playing a major role in maintaining the Th17/Treg balance in the intestine [195–197].

Changes in the composition of the gut microbiome, termed gut dysbiosis, can lead to the breakdown of immune homeostasis in the intestinal immune system in turn provoking distal abnormalities in the systemic immune and inflammatory pathways and hence making a major contribution to the development of CNS diseases characterised by demyelination [198–200]. There is an accumulating body of research suggesting that the intestinal microbiota act as environmental niches where the multiple risk factors driving the development of MS merge, thereby influencing the development of disease processes [199].

Several mechanisms have been proposed which might mediate the role of gut commensals or their antigens in driving the development of MS and other diseases of the CNS, such as the activation of myelin-specific auto-aggressive Th17 lymphocytes either via molecular mimicry or bystander activation together with the production of neurotoxic metabolites or the translocation of Gram-negative commensal bacteria containing LPS into the systemic circulation as a result of increased intestinal permeability [201, 202]. In addition, several gut microbial metabolites and bacterial products may interact with the immune system to modulate CNS autoimmunity [202, 203]. Several studies have reported the presence of increased intestinal permeability in MS patients and in mice with EAE [204, 205], which would enable the translocation of LPS into the bloodstream with consequent engagement of TLR-4 and immune activation [141]. Indeed, a recent study reported that increased intestinal permeability seemed to be present at the onset of EAE, although this finding is yet to be replicated in human studies [206].

### Leaky Gut in MDD

There is also considerable direct evidence of increased intestinal permeability and bacterial translocation into the systemic circulation in patients with MDD, which appears to make a significant contribution to levels of peripheral inflammation, O&NS and autoimmunity [207, 208]. There is also accumulating evidence that variations in the composition of the microbiota are involved in the pathophysiology of major depression as well as a range of other neuroprogressive conditions [209, 210]. Jiang and colleagues analysed faecal samples from MDD patients in the active phase of the disease and in remission and from unaffected age- and sex-matched controls utilising pyrosequencing following polymerase chain reaction methodology [211]. These authors reported increased levels of *Bacteroidetes*, *Actinobacteria* and *Proteobacteria* and reduced levels of *Firmicutes* in patients in both illness states compared with controls. Interestingly, there was also an increase in bacterial species diversity in active MDD patients compared with controls, which was not observed in patients in remission. Furthermore, reduced levels of *Faecalibacterium* were observed in MDD patients, which correlated negatively with the severity of depressive symptoms [211].

The influence of the microbiota in influencing the development of MDD appears to be multifactorial. The weight of evidence suggests that one such pathway involves the modification of bidirectional communication systems between the gut and the brain. There is now a large and accumulating body of evidence demonstrating that commensal gut bacteria have the capacity to activate several neural pathways and signalling systems in the CNS and have given rise to the concept of the microbiota-gut-brain axis which relays a variety of signals between the CNS and the gastrointestinal tract [212, 213]. While it is increasingly apparent that the optimum activity of this pathway is an essential element in maintaining intestinal and CNS homeostasis, the precise details of the mechanisms enabling such function are not fully understood although humoral, immune, neural and metabolic pathways are all considered to play a part [213, 214].

The action of translocated LPS on TLRs and the consequent activation of immune-inflammatory cascades have been discussed above and can go some way to explaining the systemically elevated levels of PICs seen in many patients with an MDD diagnosis [208]. Elevated levels of LPS can also exert a number of somewhat contradictory effects on the immune system, however, such as the activation of IDO with activation of the tryptophan catabolite (TRYCAT) pathway leading to profound immunosuppression [13]. LPS and IDO also conspire to activate Tregs, which can also lead to an immunosuppressed environment and possibly contribute to a state of T cell anergy seen in some MDD patients [215–217]. LPS activation of TLR-4 and the TRYCAT pathway in microglia, following the translocation of the antigen from the intestine, leads to a host of neuropathological consequences in animal models of depression [217, 218] and the significance of this phenomenon in the development of MDD appears to be under discussion. A summary of the similarities and differences between MS and MDD in the arena of increased intestinal permeability and bacterial translocation is provided in Table 4.

**Table 4:** Similarities in gut dysfunction between MS and MDD

Gut dysfunction	MS	MDD
Increased IgM/IgA responses to LPS/antigens of Gram-negative bacteria	Y	Y
Gut dysbiosis	Y	Y
Leaky gut	Y	Y
Leaky gut mediating immune activation, inflammatory responses or autoimmunity	Y	Y
Possible activation of the TLR-2/4 complex	Y	Y
Role of gut dysbiosis, leaky gut and bacterial translocation in the pathophysiology of the illness	Y	Y

## Evidence from Neuroimaging Studies

The association between structural brain abnormalities and the development of depressive symptomology in patients with MS has been the focus of intense empirical research for over two decades and there is an accumulating body of evidence suggesting that brain atrophy or demyelination in the frontal and temporal lobes are associated with the development of depression in at least some MS patients [4, 219, 220]. For example, Pujol and others detected a significant association between the presence of lesions in the white matter of the left suprainsular region and the occurrence depressive symptoms, which accounted for approximately 17% of the depression variance [221]. In a later study, the same team reported a significant association between the presence of depression and the existence of demyelinating lesions in the left arcuate fasciculus, which accounted for 26% of the depression variance as measured by the Beck Depression Inventory [222]. On the other hand, Bakshi and others reported that the presence and severity of depressive symptoms suffered by MS patients were predicted by the presence and extent of hypointense plaques on T1-weighted magnetic resonance images (black holes) in the superior parietal and frontal regions, as well as by lateral and third ventricular enlargement [223]. Zorzon and others examined 95 MS patients, 19% of whom qualified for a diagnosis of MDD, using T1-weighted brain MRI and reported that the presence and severity of depressive symptoms were weakly correlated with right temporal brain atrophy and right frontal lesion load [224]. Moreover, the presence and magnitude of T1 lesions in the superior frontal and parietal regions were predictive of depression in their patients [214]. In a two-year follow-up study, the same research team reported that increases in brain atrophy in the left frontal lobe were significantly greater in depressed MS patients than in those MS patients not diagnosed with depression (with a trend in the same direction for the right temporal lobe) [225]. Furthermore, these authors reported that the magnitude of atrophy displayed by patients in the right temporal lobe was a significant and independent predictor of the severity of depressive symptoms [215]. In a later cerebral MRI study, Feinstein and colleagues reported that 42% of the depression variance in their MS study cohort was accounted for by the increase in the left anterior temporal CSF volume and the increase in T2-weighted lesion volume in the left medial inferior prefrontal cortex [226].

However, while the evidence does seem to support the conclusion that structural and/or morphological changes in the frontal and temporal lobes are associated with the development of depression in at least some MS patients, many research teams in recent years have investigated potential associations between structural and/or functional abnormalities within the hippocampus and the presence of depression in individuals with MS [227, 228]. In particular, MRI studies involving patients with a confirmed diagnosis of MS have demonstrated that volume loss and abnormal morphology within the hippocampus are predictive of the presence and severity of depressive symptoms in such individuals [227–230]. This is perhaps unsurprising given that hippocampal impairment is associated with depression and with a range of cognitive abnormalities [231]. In addition, several authors have reported reduced hippocampal grey matter volume in patients with idiopathic MDD [232–234].

Significantly, while the majority of structural neuroimaging studies have revealed significant associations between the presence and or severity of depression in MS patients and various objective measures of disease burden, such as lesion load, brain atrophy and abnormalities within NAWM and NAGM; these findings have only accounted for a small percentage of the total variance of depressive symptoms in each study [4, 221, 222]. This observation suggests that these somewhat disparate findings may stem from the presence of a common underlying factor (or factors) which contributes to the pathogenesis and pathophysiology of MS and MDD [235]. This is an important point as most, if not all, these neuroimaging abnormalities are caused at least in part by neuroinflammation [236–240] and there is copious evidence that peripheral inflammation, in the guise of elevated PICs, and neuroinflammation, in the guise of activated microglia, play a causative role in the development of both illnesses as discussed above. There are also multiple lines of evidence which suggest that depressive symptoms in MS may originate as a result of synaptic impairment, derived from an abnormal production of proinflammatory molecules by activated microglia during central inflammation [166, 241, 242].

From the perspective of data demonstrating an association between hippocampal atrophy and the development of depression in MS patients, it is also noteworthy that the existence of microglial activation and other aspects of hippocampal pathology such as neuronal loss, atrophy and extensive demyelination, in at least some MS patients, has been confirmed by neuroimaging and post mortem studies in between 53 and 79% of patients recruited into studies [243–245]. In addition, among the various CNS sites involved in MS, the hippocampus is particularly vulnerable to the detrimental effects of neuroinflammation, largely because of the high density of IL-1 receptors found in that region of the brain [243, 246]. Furthermore, strong evidence confirming the presence of neuroinflammation-induced abnormalities in the hippocampus and the development of depressive symptoms in MS patients have been provided by two recent studies conducted by Rocca and fellow workers and Cosalanti and fellow workers [235, 247]. In their structural MRI and resting-state (RS) functional MRI (fMRI) study of 69 MS patients, Rocca and coworkers demonstrated a strong correlation between, on the one hand, reduced hippocampal RS functional connectivity (FC) with cortical-subcortical regions of the default-mode network and, on the other hand, increased T2-weighted lesion volume, duration of the disease, and the severity of clinical disability and depressive symptomatology [236]. These findings were supported by Cosalanti and fellow workers who utilised RS fMRI and positron emission tomography (PET) to investigate a potential relationship between the existence of neuroinflammation, hippocampal FC and the existence of depression in MS patients [224]. Importantly, these authors reported that the intensity of microglial activation, indexed by the second-generation 18-kDa translocator protein (TSPO) radioligand [^18^F]BRIII, within the hippocampus, was positively correlated with the severity of depression, indexed by the Beck Depression Inventory, while there was also a positive correlation between the hippocampal [^18^F]BRIII distribution volume ratio and the strength of hippocampal FC with prefrontal, parietal and occipital cortices [224].

## Neuroendocrine Abnormalities

### Cortisol Levels and Cortisol Awakening Response in MS

Elevated basal levels of plasma cortisol and adrenocorticotropic hormone (ACTH), together with enlarged adrenal (suprarenal) glands, have been observed in RRMS patients [248–250]. Moreover, large increases in cortisol levels have been observed in proximity to or during acute relapse characterised by a significant increase in inflammatory state [251–253]. Unsurprisingly, the increased levels of inflammatory markers in the CSF seen in patients during this phase of the illness is associated with an increase in hypothalamic-pituitary-adrenal (HPA) axis activity and a corresponding increase in the numbers of patients reporting symptoms of depression [254]. This association between increased HPA axis activity, elevated cortisol secretion and increase in inflammation may well be significant from the perspective of the pathophysiology of the illness, as increased HPA axis dysregulation in RRMS is associated with global neurodegeneration and an aggressive disease profile [255]. It is also noteworthy that basal levels of cortisol and levels of cortisol following corticotropin-releasing hormone (CRH) stimulation vary according to disease subtypes and severity of MS for reasons which are not fully understood [256]. However, one factor may be the levels and activity of CRH-secreting neurones in the hypothalamus, as increased numbers and activity of such neurones have been detected in this region of the brain in RRMS patients post mortem, possibly resulting from an upregulation in an attempt to mitigate against the presence of inflammation [252, 257]. However in severe disease, increased inflammatory lesion burden in the hypothalamus leads to reduced numbers and activity of CRH neurones and a denuded production of cortisol and thus, at least potentially, an impaired capacity to combat inflammation [258].

Several authors have investigated potential relationships between various measures of HPA axis activity and cortisol levels and the presence of depression in MS patients. For example, Gold and fellow workers reported significantly increased levels of evening cortisol in RRMS patients with depression but normal levels in patients without such symptoms compared with healthy controls [227, 259]. Similarly, Kern and others reported that elevated cortisol awakening response (CAR) levels were only observed in RRMS patients reporting symptoms of depression and noted a significant association between CAR levels and the extent of neurological disability and the presence of depression [260]. Kern and others also reported a significant association between elevated CAR values and increases in disease severity, indexed by the EDSS [261]. Briefly, these authors investigated the longitudinal relationship between CAR, extent of neurological disability as measured by EDSS and the presence of depressive symptoms in 77 MS patients over a nine-month period [261]. They noted that RRMS patients with increases in neurological disability over the relevant time-period displayed a significantly greater CAR compared with healthy participants while CAR levels in patients whose EDSS scores remained stable were not significantly different from healthy controls. This is of interest given data demonstrating an association between increased EDSS scores and increased levels of inflammatory markers and an association between increased HPA axis activity during relapse and the advent of MDD [254, 262]. However, in a departure from earlier findings by this research team, CAR values were not associated with the presence of depressive symptoms in this study nor indeed with an index of patient stress, but baseline values of EDSS and CAR were predictive of EDSS at the nine-month follow-up [250]. This latter finding is consistent with other lines of evidence which indicate that HPA axis abnormalities are associated with more severe MS and possibly even an enhanced susceptibility to developing the disease [242, 243, 247]. HPA axis hyperactivity in particular is associated with advancing disease and global neurodegeneration, possibly as a result of increases in cortisol production which may cause widespread damage in several areas of the brain such as the hypothalamus, which may go some way to explaining the associations between CAR and disease severity and/or progression and the development of MDD in patients with more aggressive disease [217, 245].

### Cortisol and CAR Abnormalities in MDD

The data regarding CAR levels in MDD are at first sight somewhat inconsistent with authors reporting elevated, lowered and normal CAR values in their study cohorts compared with healthy controls [263]. For example, Vreeberg and fellow workers reported an increased CAR in a large cohort of currently depressed middle-aged females and middle-aged females in remission [264]. Similar associations have been reported in adolescent and young adult females [265, 266]. Several other research teams have also reported elevated CAR values in medication-free patients in remission, which indicates that HPA axis dysfunction may be a trait in some individuals which confers a vulnerability to developing MDD [264, 267–269]. However, other research teams have reported a blunted CAR in young adult women and older individuals with relatively mild or moderate clinical depression [270] and a significantly reduced CAR in patients with severe depression [271]. These findings have been echoed in several other studies with patients with relatively moderate symptoms displaying an elevated CAR while patients with more severe symptoms almost invariably showing a decreased CAR both in cross-sectional and longitudinal studies [272, 273]. The reduced CAR in severe depression indicates HPA axis hypofunction possibly as a result of CRH receptor downregulation as a result of chronically elevated levels of PICs rather than the reduced numbers and activity of CRH neurones [264] seen in severe RRMS [258].

### Glucocorticoid Receptor Resistance in MS

There is accumulating evidence, largely from cross-sectional studies, that glucocorticoid (GC) receptor (GR) sensitivity, as determined by proliferation of T cells and PIC production, is significantly impaired in RRMS patients compared with healthy age- and sex-matched control subjects [274, 275]. This phenomenon is of importance as the activity of these receptors mediates the response of corticosteroids released by HPA axis activation and is essentially the effector of the neuroendocrine anti-inflammatory response (reviewed [276]). This is clinically significant as GR resistance is associated with non-responsiveness to corticosteroid therapy used in an attempt to reduce the time spent in relapse [277, 278] (reviewed in [279]).

Perhaps unsurprisingly, several polymorphisms affecting GR sensitivity, such as N363S, ER22/23EK and *Bcl*1 C/G, have been associated with more aggressive MS as assessed by EDSS and MRI [280, 281]. However, a recent larger, multi-ethnic study found no association between the presence of these polymorphisms and illness severity and that non-genetic factors such as the presence of inflammation and elevated PICs were the most important cause of GC resistance in RRMS patients [282]. This conclusion is supported by other lines of evidence which indicate that the GC sensitivity of T cells can be modulated by PICs and other inflammatory molecules [283–285]. There are also data suggesting that GC resistance is more pronounced during relapse than during remission [252, 286].

### GR Resistance in MDD

The weight of evidence suggests that GR resistance and abnormalities in GR functioning occur in approximately 80% of MDD patients and are held to be the most reproducible biological measures associated with the illness [287–289]. These are important findings as there is accumulating evidence that PIC- and/or p38 MAPK-induced GR resistance may be a major driver of the increased inflammatory response system observed in many MDD patients which in turn is responsible for HPA axis hyperactivity seen in many individuals afforded this diagnosis [290–292]. This phenomenon has important implications from a treatment perspective, as GR resistance secondary to increased inflammation is associated with resistance to antidepressants, and there is some evidence that GR resistance must be overcome before a patient enters remission [278, 280, 281] The significance of GR resistance is further emphasised by the presence of data demonstrating a significant association between the development of GR resistance in patients and the advent of depressive symptoms [293]. Chronic stress and the resultant upregulation of inflammatory pathways as part of the conserved transcriptional response to adversity also appear to be causes of GR resistance [294, 295]. This is of interest as this stress-induced inflammatory response in combination with diminished GR responsiveness is considered to underpin the pathogenesis of MDD [296]. Moreover, the conserved transcriptional response to physical adversity is also activated by a range of perceived non-physical or symbolic threats which in part depend on within-patient factors [285]. These are intriguing observations as they allow for a contribution from psychosocial factors to the inflammatory burden of an individual MS patient and possibly to the severity of the disease experienced and indeed to the existence, or otherwise, of depressive symptoms. Finally, impaired GR function makes effector T cells resistant to chemokine-induced recruitment into the CNS [297]. This is of importance as T lymphocyte recruitment into the CNS plays an important role in maintaining cognitive function, neurogenesis and synaptic plasticity in an environment of neuroinflammation [298, 299] and thus GR resistance may contribute to the severe cognitive dysfunction and impaired neurogenesis seen in many people afforded a diagnosis of MDD.

## Therapeutic Implications

### Desipramine

Given the arguments made in this paper, it would seem reasonable to propose that a molecule which has antidepressant, antioxidant and anti-inflammatory actions might have therapeutic actions in MS. Desipramine, which is an active metabolite of the tricyclic antidepressants imipramine and lofepramine, is such a molecule.

The antidepressant activity of desipramine, which inhibits the central reuptake of noradrenaline (norepinephrine) and, to a lesser extent, serotonin, has been well established since the mid-1960s [300, 301]. The combined plasma concentrations of desipramine, which is a demethylated metabolite of the “gold standard” tricyclic antidepressant imipramine, and is also known as desmethylimipramine (formally, 10,11-dihydro-5-[3-(methylamino)propyl]-5H–dibenz[b,f]azepine monohydrochloride), and imipramine have been found to show an approximately linear relationship with clinical antidepressant response, at doses higher than a minimal plasma threshold below which such improvement is not seen [302–305]. Indeed, it has been suggested that the parent antidepressants imipramine and lofepramine act as prodrugs of desipramine [305].

The antioxidant and anti-inflammatory actions of desipramine (unlike imipramine) are also well established, with a number of studies demonstrating its protective effects against oxidative stress. Desipramine has been shown to have antioxidant and anti-inflammatory actions in experimentally induced colitis in rats, with the drug attenuating the severity and extent of tissue damage, reducing myeloperoxidase activity (in a dose-dependent way), increasing the level of reduced glutathione in colonic tissue and diminishing PIC levels [306]. In olfactory-bulbectomised rats, desipramine has been shown to reverse the usual depressed neutrophil phagocytosis associated with this operation, to shorten the time to the start of phagocytosis, and to reverse the decreased activity of glutathione peroxidase [307]. In a murine model of chronic fatigue syndrome (myalgic encephalomyelitis), desipramine has been found to attenuate oxidative stress, as well as decreasing the immobility time, increasing locomotor activity and diminishing anxiety; in particular, there was a dose-dependent increase in reduced glutathione, a dose-dependent increase in catalase, and a dose-dependent reduction in nitrite levels [308]. Similarly, desipramine has been shown to attenuate oxidative damage and to restore mitochondrial enzyme activities, including of Complexes I, II and IV of the ETC, in a murine ischaemia/reperfusion injury model of transient global ischemia [309]. Similarly, desipramine has been shown to have antioxidant and anti-inflammatory actions in a murine model of carrageenan-induced inflammation [310].

Given the importance of HPA axis and hippocampal functioning in MS, described earlier, it is germane to mention a controlled murine study by Bravo and colleagues in which the effects of chronic stress were studied [311]. Chronic stress was associated with increased body mass, which was prevented by desipramine; a large increase in serum corticosterone levels, which was prevented by desipramine; anhedonic behaviour, prevented by desipramine; increases in measures of learned helplessness, prevented by desipramine; and increased immobility, which was significantly reduced by desipramine [305]. It has been hypothesised that phosphorylation of extracellular signal-regulated kinase (ERK)1/2 might mediate antidepressant action; in this study, hippocampal levels of phospho-ERK1/2 (P-ERK1/2) were assayed and indeed the ratios of P-ERK1/ERK1 and P-ERK2/ERK2 were found to be elevated in the group of rats exposed to chronic stress, but this effect was reversed by desipramine [305]. Finally, chronic stress and GCs have been associated with decreased expression of brain-derived neurotrophic factor (BDNF) [312]. In the study by Bravo and colleagues, chronic stress was indeed associated with a reduction in *BDNF* mRNA and this was prevented in the CA3 hippocampal region by desipramine [305]. Thus, there is good evidence that desipramine has neuroprotective actions, including in the hippocampus.

### Clinical Trial

While desipramine is not available for oral administration, its parent prodrugs lofepramine and imipramine are readily available in such a formulation (although, at the time of writing, lofepramine is not available in certain countries, including the United States of America and Australia). In 1996, the late Cari Loder, who herself suffered from MS, published a book describing the possibility, based on her serendipitous experience, that MS symptoms might improve with treatment using lofepramine, L-phenylalanine and vitamin B_12_ [313]. A randomised, placebo-controlled, double-blind trial of this combination subsequently took place in the United Kingdom; the lofepramine-based treatment was indeed found to be effective in relieving MS symptoms, with benefits being noted within a fortnight to a month [313, 314]. The improvement was statistically significant but the authors of the study cautioned that the improvement was clinically small and that further research was needed to confirm and explore its significance [314].

No further clinical trials in MS involving lofepramine have taken place. However, a subgroup of the MS patients taking part in the above study underwent MRI scanning of the brain by Puri and colleagues at baseline and six-month follow-up. Using a highly accurate method for quantifying changes in ventricular volume (an index of cerebral atrophy) developed by the same group [315], it was found that the mean lateral ventricular volume increased far less in the treated group than in the untreated one; moreover, in the treated group the ventricular size change correlated with changes in Gulick MS-related symptoms scale scores and Gulick MS-related activities of daily living scale scores [316]. Furthermore, in the treated group, there was a significant reduction in the number of focal lesions on the T1-weighted scans [316].

## Summary and Conclusion

Elevated O&NS and compromised antioxidant defences in the periphery and CNS are strongly implicated as a causative element in the pathophysiology of MS and MDD. Evidence of a causative role includes an established correlation between levels of oxidative stress and number of relapses and increases in levels of O&NS prior to relapse in MS and correlation between O&NS levels and severity of illness in MDD.

Imbalances in the production of pro- and anti-inflammatory cytokines are seen in the serum and CNS of both illnesses and it is noteworthy that the concentrations of one or more cytokines correlate with the severity of both illnesses along several different dimensions. For example, levels of TNFα in the periphery are predictive of relapse and correlate with disability and fatigue, while levels of IL-1 are predictive of a poor prognosis and cortical lesion load. It is also noteworthy that peripheral levels of IL-6 and CRP differentiate MS patients with depression from those free of depression. This is perhaps unsurprising as levels of peripheral inflammation correlate with the severity and chronicity of MDD. The presence of abnormally high levels of IL-17 in the periphery and CNS would appear to clearly differentiate patients with MS from those with a diagnosis of MDD. This highly inflammatory and tissue-damaging cytokine is generally produced by activated Th17 T cells and to a lesser extent B cells and intestinal dendritic cells and these differences in cytokine population between the two illnesses might be a reflection of the fact that the source of cytokine production in MDD patients appears to be M1-polarised macrophages, neutrophils, anergised T cells and the NLP-3 inflammasome while cytokine production in MS is primarily driven by activated Th1 and Th17 lymphocytes and effector B cells, although recent evidence suggests DAMP-mediated inflammasome activation in this illness as well.

Given the presence of excessive inflammation and oxidative stress, the presence of widespread mitochondrial dysfunction in the brain and periphery of sufferers of both illnesses is unsurprising. The nature of mitochondrial dysfunction largely confined to enzymes of the ETC is also very similar in MS and MDD patients. This is strongly suggestive of the detrimental effects of chronically elevated NO and peroxynitrite. It does seem likely that mitochondrial dysfunction does make an independent contribution to the pathophysiology of both illnesses both as a result of ATP depletion and increased production of ROS. The exact origin of chronic O&NS, immune-inflammation and mitochondrial dysfunction are very difficult to determine given their complex interrelationship but, once commenced, established biochemical relationships predict the development of a self-amplifying pathology. This may well be a reason why simple antioxidants have met with limited success as treatment options while preparations targeting mechanisms driving the generation of oxidative stress, inflammation and mitochondrial dysfunction appear to be more promising.

There is now a considerable body of evidence demonstrating abnormalities in microglial activation and/or function in MS and MDD. Microglia can be activated as a result of chronic peripheral inflammation and activation of these glial cells plays a major role in the pathogenesis and the pathophysiology of MS largely as a source of cytokines, O&NS, inflammatory mediators and neurotoxic TRYCATs. The neurotoxicity and polarisation pattern of microglia in MS are heavily determined by bidirectional interactions between activated encephalogenic Th17 T cells from the periphery which is not a feature in MDD patients. Epigenetic dysregulation in terms of abnormal histone acetylation and miRNA production is clearly an element in the pathology induced by microglia and the extreme neurotoxicity of Th17 T cells seen in MS which once again does not seem to be the case in MDD. The source of Th17 T cell activation in MS is not fully understood but may result from inflammation-induced dysbiosis and breakdown of intestinal homeostasis. The nature of microglial pathology in MDD appears to be different from that seen in MS and would appear to involve the impaired function of many of their CNS housekeeping roles such as maintaining synaptic plasticity, learning memory and other aspects of cognitive functioning.

Increased inflammation could underpin the increased BBB permeability, intestinal permeability and bacterial translocation seen in MDD. While increased intestinal permeability can be a cause of dysbiosis; the quite distinct patterns of dysbiosis seen in MS and MDD indicate another origin for these observations. The advent of depressive symptoms in MS appears to be associated with the levels of brain atrophy and demyelination in NAWM and NAGM in the frontal and temporal lobes, indicating that such symptomatology is a consequence of disease process. The relationship between altered morphology, reduced volume and lesions in the hippocampus caused by inflammatory mediators released by activated microglia and the development of depressive symptoms would also support such a conclusion. However several lines of research also indicate that psychosocial factors could make a contribution to the pathogenesis of depressive symptoms in MS, most likely by increasing the level of peripheral inflammation and subsequent neuroinflammation.

Theoretically, the antidepressant lofepramine and, in particular, its active metabolite desipramine, may be beneficial not only for the depressive symptomatology but also for the neurological symptoms in MS. One clinical trial has been carried out thus far with, in particular, promising MRI findings.

In conclusion, multiple lines of evidence suggest that many abnormalities common to MS and MDD likely have their origins in the presence of chronic inflammation and concomitant oxidative stress and individual differences could be a product of disparate genetic, epigenetic and environmental influences. However, when the evidence is considered as a whole, the view that MDD exists as an entirely separate nosological entity from “MS-associated MDD”, which is associated with a characteristic array of empirical abnormalities, would appear to be reasonable.
